# The thorny complexities of visualization research for clinical settings: A case study from genomics

**DOI:** 10.3389/fbinf.2023.1112649

**Published:** 2023-03-29

**Authors:** Emilia Ståhlbom, Jesper Molin, Anders Ynnerman, Claes Lundström

**Affiliations:** ^1^ Department of Science and Technology, Linköping University, Linköping, Sweden; ^2^ Sectra AB, Linköping, Sweden; ^3^ Center for Medical Image Science and Visualization, Linköping University, Linköping, Sweden

**Keywords:** visualization, bioinformatics, genomics, next-generation sequencing, copy number variant (CNV), visual analytics (VA), cancer, rare diseases

## Abstract

In this perspective article we discuss a certain type of research on visualization for bioinformatics data, namely, methods targeting clinical use. We argue that in this subarea additional complex challenges come into play, particularly so in genomics. We here describe four such challenge areas, elicited from a domain characterization effort in clinical genomics. We also list opportunities for visualization research to address clinical challenges in genomics that were uncovered in the case study. The findings are shown to have parallels with experiences from the diagnostic imaging domain.

## 1 Introduction

Readers of this journal are likely to concur that research on visualization for bioinformatics data is both important and challenging. In this perspective article we will discuss a subarea where additional complex challenges come into play: visualization methods targeting clinical use. In our experience, these thorny complexities are particularly pertinent in genomics.

From several aspects, biomedical visualization research has been thoroughly mapped out. In terms of application areas, Garrison et al. (2022) provide a comprehensive overview. In terms of articulating the scientific discipline as such, Raidou et al. (2020) describe many key aspects. We argue, however, that the complexities of targeting clinical work constitute another fundamental but less recognized aspect of this discipline, complementary to visualization research targeting scientists.

A main contribution of this article is to articulate the challenge areas arising when visualization research in bioinformatics targets clinical use. The four challenge areas were identified through a case study, a domain characterization effort in clinical genomics. Another contribution is the agenda for visualization method development opportunities uncovered by the case study. Finally, we will put our findings in a greater context, where we find strong parallels with visualization for diagnostic imaging.

## 2 Background

### 2.1 Clinical workflow description

In this section we will briefly walk through the clinical workflows that constitute the background of this perspective article. Around the world, precision medicine initiatives are underway that can be expected to greatly increase the use of DNA sequencing in clinical routine, such as using broad panels as the norm for oncology patients.

DNA sequencing is performed by request from the patient’s treating physician. The output data from sequencing is interpreted by a variant analyst, who relates their findings in a report to the ordering physician. Interpreting the data entails finding variants that are relevant to the patient’s disease and relating said variants to previous knowledge. The variant analyst needs to navigate multiple gigabytes of data per case, and sort through an assortment of knowledge databases. Thus, the task of efficiently reaching insights through data-intensive analysis fully maps to the core value proposition of visualization methods.

**FIGURE 1:**
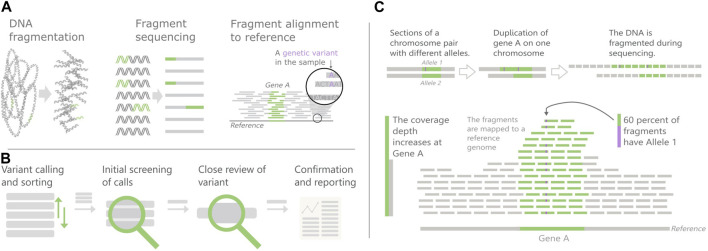
Overview of Next-generation sequencing (NGS) and Copy number variants (CNVs). **(A)** The NGS principles **(B)** Variant review pipeline, **(C)** CNV detection in NGS data. CNVs cause an increase in the number of fragments mapped to their area, which can skew the fraction of segments with different alleles.

The most common method for DNA sequencing is next-generation sequencing (NGS), where the sample DNA is broken into fragments before being sequenced (Figure 1A). The fragment sequences are then mapped to a reference genome based on similarity, to determine their most likely original coordinates. The vast amount of data is handled through use of callers, software algorithms that find suspected variants, calls, in the sequencing output data. Simply put, the work of the variant analyst is to review the calls and report conclusions relevant for treatment decisions to the physician (Figure 1B).

This perspective article will use analysis of copy number variants (CNVs) as a case study. A CNV is an increase or decrease in the number of copies of a large section of the genome. Current research shows that CNVs are important in cancer development as well as for some hereditary diseases, but little is still known about their full impact. They are also complicated to find in NGS data since they are longer than the DNA fragments.

### 2.2 Visualization for genomics

Challenges and opportunities relevant for visualization in clinical genomics has been discussed in previous work. Handling variants of unknown significance is one such challenge (Biesecker, 2012; Hellwig et al., 2019), as is other types of decision support design (Yaung and Pek, 2021; Kulchak Rahm et al., 2021). Effective reporting is another area in focus, both to ordering physician and to patients (Biesecker (2012); Wynn et al., 2018; Sanderson et al., 2019). Tools for collaboration and knowledge sharing between geneticists have also been studied (Liang, 2017).

A good entry point to NGS visualization methods is the survey and taxonomy presented by Nusrat et al. (2019), which was also the base for visualization framework efforts (L’Yi et al., 2022; Pandey et al., 2022; L’Yi and Gehlenborg, 2022). Other previous efforts have proposed methods targeting specific genomics analysis tasks (O’Brien et al., 2010; Ruddle et al., 2013; Ferstay et al., 2013; Reber et al., 2013). Some of these methods partially target an everyday clinical scenario, whereas some do not, but many components and ideas are nevertheless promising to include in future clinical solutions.

Efforts mainly residing in the bioinformatics research community often include tools to visualize genomics data as well. As our case study centers on CNV analysis, CNV visualization tools are the most relevant here (Tebel et al., 2017; Sante et al., 2014; Macnee et al., 2022; Chandramohan et al., 2021; Markham et al., 2019; Ramesh et al., 2022; Ma et al., 2015; Zhou et al., 2021; Chanwigoon et al., 2020). Many tools target cohort analysis and other types of research-only use. A common trait for the development of these tools is that the respective design process is tightly connected to the specific needs and requirements of the creator’s own organizations. Broader analyses of domain challenges and in-depth end-user evaluations are not in focus, making it difficult to draw conclusions on how well the tools would fit other scenarios and organizations.

## 3 Case study

The empirical examples of the challenge areas, discussed below, stem from a case study on CNV visualization needs. In the first phase of an ongoing research effort to design genomics visualizations for the clinic, based on a design study approach (Meyer and Dykes, 2020), we performed a domain characterization based on interviews. We conducted three in-depth semi-structured interviews with predetermined interview guides as scaffolds, with domain experts (bioinformaticians and geneticists) who work with analyzing NGS data for diagnosis of inherited disease. All three domain experts had experience from all steps in the variant review pipeline of Figure 1B. In addition, informal discussions were held with genomics laboratory personnel (including technicians, geneticists, and bioinformaticians) from both oncology and clinical laboratories. In total thirteen participants across four different hospitals were consulted. We focused on inherited disease as reviewing CNVs in clinical routine today mainly is done within that area in the hospitals we visited.

**FIGURE 2:**
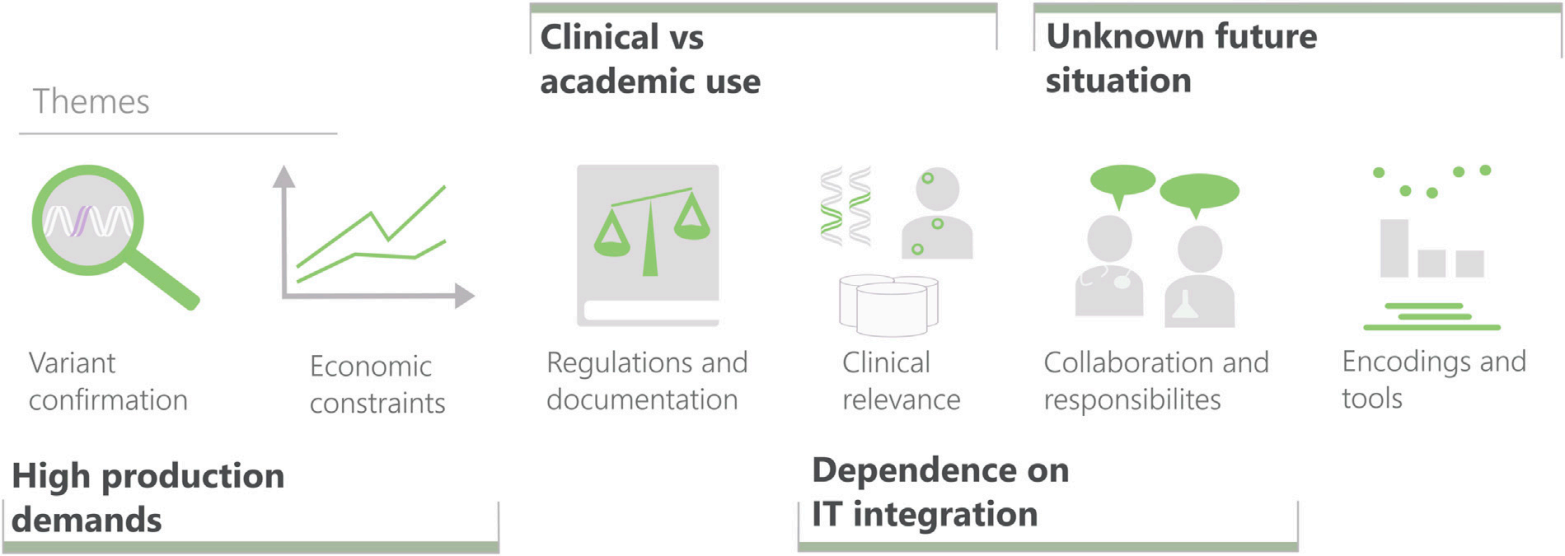
The six themes characterizing variant interpretation created from interview data and the four elicited challenge areas for visualization research in clinical settings (bold), including their relation to the themes. Themes, from left to right: Variant confirmation (mainly ruling out false positives). Effects of limited time and resources. Constraints imposed by laws and regulations (ranging from IT security to validation of laboratory processes). Evaluating the clinical relevance of a variant. The interplay between the professions involved in the diagnostic work. Encodings and tools used for displaying CNVs.

The structure of the deep interviews was as follows. After an introduction to the study, the participants were asked to describe the situations in which they do CNV analysis. The interview then moved to questions to drill down into details about analysis tasks, data, existing tools, reporting, and wish lists for the future. The participants were encouraged to explain through case walk-through, while speaking about the performed actions out loud. The final part consisted of getting feedback on paper sketches on visualization designs. The informal discussions followed shorter interview guides or were led primarily by the participant. We used transcriptions and thematic encoding (Braun and Clarke, 2006) to analyze the material, resulting in six themes summarized in Figure 2.

## 4 Challenges and opportunities for visualization research

The focus of this perspective article is to outline generic challenges for clinical visualization research. With that objective, we analyzed the interview findings to regroup them into such challenge areas. The four areas elicited will be described next. For each area, a first paragraph articulates the respective visualization research challenge, which then is followed by underpinning examples from the genomics case study. The relations between themes and areas are given in Figure 2. The case study also uncovered distinct unmet needs that form an agenda for visualization method development in this domain, which is outlined in Section 4.5.

### 4.1 High production demands

The first challenge area in clinical settings is the demand of high throughput. Economic pressure in healthcare is severe, leading to a mindset where any activities aside from those directly affecting patient management typically are discarded. In essence, the challenge here lies in giving the productivity demand a sufficiently dominant weight, which is in conflict with a visualization researcher’s natural ambitions to convey as much insights from the data as possible.

Several findings in the Variant confirmation and Economic constraints theme of the case study illustrate the challenges of clinical work being a high-production workflow. First and foremost, a recurring observation is: Time constraints limit the breadth of which calls to review and the depth of review for each variant called. Only some calls are investigated, where the trade-off is made between the risk of missing something essential and the waste of spending time on unnecessary analysis. The decision on how to limit breadth and depth leans heavily on the time available. Participants expressed a desire to focus their attention only on the most promising calls, and requested better methods for ranking the calls.

As further exemplified in the descriptions of variant confirmation work, a particularly important aspect is that the list of potential CNVs contains many false positives and is typically too long to go through thoroughly. To work effectively, the analysts rely on CNV caller methods for filtering and sorting the calls, but the quality of callers is a common source of frustration. One participant describes it as:

”Either you have very low sensitivity or […] you’re drowning in false positives”.

### 4.2 Clinical vs. academic use

A related area is the difference between clinical and academic work, which includes more aspects than the difference in production pace discussed above. This is particularly articulated in genomics, an area characterized by academic operations being very influential on clinical use. This prerequisite poses a risk that visualization methods are inadvertently developed for a research setting, and that it can be difficult to realize what advances are needed to reach clinical effectiveness.

An overarching observation, essentially corresponding to the entire Clinical relevance theme, pinpoints this challenge area: Only findings that have clinical relevance for the patient’s symptoms are investigated and reported. In other words, work that is spent eliciting information that cannot be used to guide the patient’s treatment is considered as wasted time. This type of usefulness emphasis is represented also in other matters, including diverging opinions among the participants regarding whether whole-genome sequencing is worth the extra cost (including the additional review time).

Another area of contrast is represented by findings in the Regulations and documentation part of the case study. In clinical settings, the documentation of the review is dictated by regulatory demands. The responsibility is summarized as follows by one of the respondents:

”We have to make sure that it works from sample arrival until report submission.”

When asked about how documentation of the interpretation process was solved in practice, participant’s answers referred to manual activities such as keeping intermediary results in their heads or writing on paper what to follow up on later in the process.

The regulatory demands also refer to validation of both the sequencing and review process. While scientific rigor also requires good control of genomics operations, for clinical work, the moral and legal responsibility for the welfare of actual patients constitutes a higher bar. In some cases, the academically developed CNV detection methods are not validated for clinical use, which means that any findings must be verified with another technique.

### 4.3 Unknown future situation

Empirical evidence for successful visualization designs is typically gathered through user studies. In this respect, a challenge applicable to most visualization research is that a new tool will have an unfair disadvantage compared to the existing tool which is well known to the end user. The challenge is, however, much greater when future workflows are unknown and not even the end user knows what the future situation will be like.

The challenge area of designing visualizations for unknown future scenarios is highlighted by findings regarding roles and competence in the Collaboration and responsibilities theme. In several ways, participants expressed that current practices are in need of improvement. One sign of lacking maturity is that the competence mix and work practices were different across the sites. Moreover, one respondent highlighted the need for more and deeper collaboration across professions as genomic testing becomes more complex. However, the main finding pointing to the necessity of future changes to work descriptions and role responsibilities is that several respondents underlined the challenge of varying experience in the laboratory team.

Knowledge of laboratory procedures and understanding of the bioinformatics software creates an awareness of their limitations. For example, novice variant analysts might trust pathogenicity prediction software too much. One participant expressed concern regarding errors when designing the sequencing process or interpreting its results, that could be made by an analyst or physician with less experience.

Further illustration of this challenge area comes from the Encodings and tools theme. Several sites use result presentation conventions from microarray visualization, the previously predominant genomics testing method, to address varying levels of experience. For example, one site employs base pair binning to display copy number in scatterplots instead of the bar chart of coverage depth. While much detail is lost, it decreases the need for training personnel to work with a new data representation.

Thus, there is currently a disconnect between the competence of the roles in the process, and what at least the most competent users believe corresponds to high quality care. Time will tell what the situation will converge to (in terms of competence development, new roles, adapted workflows, etc.), but it is clear that a visualization researcher needs to target scenarios that are not fully in place today.

### 4.4 Dependence on IT integration

The fourth challenge area is the difficulty to isolate clinical tools from each other. The dependence on the integration with existing IT systems, such as the electronic health record or laboratory information system, will have great impact on the user experience of any visualization tool. Thus, a user evaluation of a novel visualization application in an isolated setup may be of little relevance for usefulness in an actual, integrated clinical setting.

IT integration aspects often appeared in the interviews. With regards to the Clinical relevance theme, several participants expressed that they needed to search in many different places to get a clear picture of current knowledge of their variant. Some sources are used to investigate the function of the gene, some contain variant occurrence frequency and pathogenicity, and others focus only on one variant type or disease. This is perhaps best summarized by the following statement:

”I have a thousand tabs open at the same time.”

Joint work across sites also adds IT complexity, as interviewees reported in the Collaboration and responsibilities theme. One site relies on another site for some of their bioinformatics, for example. The integration area also connects to the challenges of targeting an unknown future, as the respondents expressed desire to include further systems not yet available due to legal concerns about cloud services.

### 4.5 Research opportunities for genomics visualization research

Apart from informing the challenge area descriptions, many of the domain characterization findings also describe current pain points that constitute opportunities for visualization research in terms of method and application development.

In the variant call screening step, the task of narrowing down the call list to a few candidates for further scrutiny is described as time-consuming if manual, or error-prone if automated. A visualization opportunity in this context is condensed glyphs or other representations to give pre-attentive cues to allow the analyst to skip irrelevant list entries, at subsecond pace.

The multiscale characteristic of variant review offers possibilities for tailored visualization methods. Semantic zoom concepts, where information items persist but become more granular when drilling down, appear as a promising approach. The large number of sources that needs to be involved in the variant review points towards linked views solutions, but at a particularly challenging level.

In several analytic steps, effective comparative visualization designs will be needed, as the genomic output needs to be compared to other individual cases, local cohorts, and larger populations. A research target would be to address this need by developing condensation methods for difference graphs. Another aspect that will need to permeate many visualization components is uncertainty awareness, a major factor for instance in determining clinical relevance.

With regards to the need for documentation, it would be interesting to study how well visualization provenance methods could tackle this, in combination with automatic summaries of key events. Along similar lines, an interesting research target would be methods for report generation that captures the necessary conclusions in the background without explicit user entries.

## 5 Discussion

We have presented our insights on how an ambition to do visualization research targeting clinical needs will entail additional challenges. We hope that these perspectives can be useful for future efforts towards this, in our view, highly motivated type of medical visualization research. Insights from visualization researchers working with scientists from other domains were described by Beyer et al. (2020). Notably, in that context the challenge areas presented here are not brought up, again pointing to a distinct difference in working in the clinical setting compared to a scientific usage scenario.

The conclusions from our domain characterization in genomics are well aligned with our experiences from many years of similar work in radiology and pathology. High production demands were confirmed in our work to characterize visual analytics in radiology, where time pressure was seen by radiologists as the most challenging aspect of all (Lundström and Persson, 2011). This means that tools entailing even a minor additional workload are unlikely to be adopted even if there are other benefits. The current situation of rapid evolution of clinical genomics practices can be related to the digitization of pathology imaging, that for us posed the research challenge of designing visualizations for a not yet existing user group (Molin et al., 2015). The need to account for productivity demands and IT integration has been underlined also in the area of clinical decision support in tumor boards (Müller et al., 2021).

With regards to clinical vs. academic use, there is a parallel to the current focus of artificial intelligence (AI) implementation in diagnostic imaging. There is a disconnect between the isolated sandbox of typical AI studies and the actual clinical workflows (Adler-Milstein et al., 2021). Moreover, the performance evaluations during method development are not sufficient, and validation in the actual clinical environment is necessary (Daye et al., 2022). The final challenge area about the need for integration with existing systems also turns up in diagnostic imaging AI, as it is highlighted as a key success factor for clinical implementation (Daye et al., 2022).

The perspectives described in this article can also be put into a bigger picture. The traditional mindset for the process of making research impact in healthcare is to go from identifying knowledge gaps in medicine, carrying out basic research addressing them, then carrying out translational research efforts, followed by clinical implementation. The model for visualization research towards clinical use is a different one, that one could call *healthcare‐native research*. Here, the clinical prerequisites do not come in only in a translational phase, but constitute the origin of the research agenda and permeates the method development throughout.

In conclusion, the complexities of visualization research for clinical settings are thorny—but challenges are also inspirational for making advances. We hope that many will agree with us that the many opportunities for visualization to have impact in the bioinformatics areas of healthcare should attract much scientific interest in years to come.

## Data Availability

The original contributions presented in the study are included in the article, further inquiries can be directed to the corresponding author.
